# Development of a portable smart Glucometer with two electrode bio-electronic test strip patch based on Cu/Au/rGO/PEDOT:PSS

**DOI:** 10.1038/s41598-023-36612-4

**Published:** 2023-06-12

**Authors:** Masoomeh Monfared Dehbali, Milad Farahmandpour, Samaneh Hamedi, Zoheir Kordrostami

**Affiliations:** 1 Department of Electrical Engineering, Shiraz University of Technology, Shiraz, Iran; 2 Research Center for Design and Fabrication of Advanced Electronic Devices, Shiraz University of Technology, Shiraz, Iran

**Keywords:** Biophysics, Biotechnology, Engineering

## Abstract

Today, the importance of blood sugar monitoring in diabetic patients has created a global need to develop new glucometers. This article presents the fabrication of a portable smart glucometer for monitoring blood glucose with high sensitivity. The glucometer employs a bio-electronic test strip patch fabricated by the structure of Cu/Au/rGO/PEDOT: PSS on interdigitated electrodes. We demonstrate that this structure based on two-electrode can be superior to the three-electrode electrochemical test strips available in the market. It has good electro-catalytic properties that indicate high-performance sensing of blood glucose. The proposed bio-electronic glucometer can surpass the commercial electrochemical test strips in terms of response time, detection range, and limit of detection. Electronic modules used for the fabrication of smart glucometers, such as a power supply, analog to digital converter, OLED screen, and, wireless transmission module, are integrated onto a printed circuit board and packaged as a bio-electronics glucometer, enabling the comfortable handling of this blood glucose monitoring. The characteristics of active layers biosensors were investigated by SEM, and AFM. The glucometer can monitor glucose in the wide detection range of 0–100 mM, the limit of detection (1 µM) with a sensitivity of 5.65 mA mM^−1^ and excellent sensing performance such as high selectivity, high reproducibility, and good stability of fabricated test strips. With 11 human blood and serum samples, the glucometer demonstrated high clinical accuracy with the best value of RSD of 0.012.

## Introduction

Biosensors are a basic component in the field of medical diagnosis for various disorders. The majority of the common biosensors measure the dosage of various biochemical analytes in a watery environment^1^. The rapid progress of bio-electronic has produced different medical methods that accurately detect various biological disorders. Diabetes mellitus (DM) is one of the common chronic diseases due to an imbalance of the body's blood glucose values. According to a report National Diabetes Federation (IDF), 425 million diabetic people suffered from these diseases (type I and II) worldwide in 2017^2^. DM is the main cause of mortality and morbidity in each country and a common disease seen among chronic conditions^3^.

In recent years, a wide variety of researches have been done on the glucose sensing methods, such as optical, electrochemical, and field effect transistor (FET) devices^4,5^. At present, electrochemical glucometers are used to monitor glucose concentrations in the markets.

In general, graphene, carbon nanotube, metal oxides such as CuO, ZnO, Fe_2_O_3_, TiO_2_, Ag_2_O, SnO_2_, and polymers such as ethylene glycol, phenylboronic acid have been fabricated for glucose sensing^6–19^. Among the above materials, graphene is commonly used for glucose sensors because of its low cost, and high electrocatalytic properties for detecting glucose^20^.

Graphene oxide (GO) is the most common candidate as a high-performance two-dimensional material for selective and sensitive glucose sensing due to its electron transfer capability, bio-molecular affinity, high electrochemical activity, and chemical stability, good physical properties, high ratio of the surface to the volume, excellent mobility, and high flexibility^21^. Its application in glucose sensing, solar cells, gas sensors, transistors, photo-detectors and batteries have been studied^22–29^.

Po3,4-ethylene dioxythio pheneene:polystyrene sulfonate (PEDOT:PSS) is a very demanding polymer because of its extraordinary characteristics and key advantages such as enhanced electrical conductivity, electrical stability^30,31^, and flexibility^32–36^. In recent years, research on PEDOT: PSS has attracted much attention^37–39^. It can be used with many solution-based fabrication processes, such as deposition methods like drop-cast and printing methods like ink-jet printing and screen printing^40–43^. The studies on PEDOT: PSS-based sensing applications are gas sensors^44,45^, electrochemical sensors^45–47^, temperature sensors^48^, and electronic switching^49^. PEDOT: PSS has been used to improve the sensing performance, and the structural instability of nano-materials^49^. This conductive polymer is usually used in the form of a hybrid with metal oxides and carbon nanostructures.

Rafiq Ahmad et al. reported a nanostructured CuO–ZnO hybrid based on glucose biosensor. The externally modified CuO has the potential to enhance excellent electrochemical activity^18^. Jung, Ahmad et al. used NiO QDs-ZnO NRs on Polyimide substrate based on glucose sensors. NiO improved biosensor sensitivity^50^. Ensaf et al. studied silver nanoparticles decorated with MWCNTs for electrochemical glucose sensing to improve their performance^51^. Brianna Barbee et al. reported a wafer-scale fabrication of Cu–Ni thin films via the RF magnetron sputtering method for glucose detection. The fabricated wafer showed excellent electrocatalytic properties for glucose oxidation. The rGO/PEDOT: PSS is a composite material that has the potential to improve signal transduction for better detection.

As mentioned above researchers reported, they used electrochemical methods that suffered the complicated process of glucose sensing. The electrochemical biosensors use three electrode system consists of a working electrode, counter electrode, and reference electrode. This method suffers from the complicated process of glucose detection, large dimensions, the need for a potentiostat instrument and no good stability^52^. Another method for biosensing are bio-field effect transistors (bio-FETs). Most of the biosensors are based on FET as the gate is exposed to the solution and gate voltage is applied through an Ag/AgCl reference electrode. This method has some disadvantages that their costs are high and their efficiencies are lower than electronic devices. This paper solves the electrochemical method challenge with the concept of a bio-electronic test strip patch on the smart glucometer presentation with a novel design. Bio- electronic-sensors are smart tools in microelectronics that have opened up possibilities in the body as implantable, invasive, minimally invasive, and wearable biosensors. The using bio-electronic technology is better and more sensitive than available electrochemical glucometers technology that has been utilized in the market for glucose monitoring and also can be used in wearable applications to measure continuous and real glucose values and also integrated with other biological microchips.

In this study, we have fabricated two types of sensors based on PEDOT: PSS and rGO/PEDOT: PSS and compared their electrical responses. We fabricated a portable smart glucometer for blood glucose monitoring via a test strip fabricated by Cu/Au/rGO/PEDOT:PSS on interdigitated electrodes (IDE) to obtain high sensitivity. The glucometer integrates an OLED screen for glucose display in the unit of mg/dl, a microcontroller for signal processing, and a Bluetooth module for wireless transmission integrated on a printed board (PCB). A bio-electronic glucose sensor patch connects to the glucometer for blood glucose sensing. The power source of the whole glucometer system is a 9 V battery. The extracted glucose molecules are then detected via the bio-sensor.

The novelty of the work lies in the morphology of rGO and PEDOT:PSS as active materials that has caused achieving high sensitivity and performance. A special morphology from rGO/PEDOT:PSS (vertically grown nanorods with rGO sheets) has caused increased surface to volume ratio and has improved the sensor performance. Another novelty of the proposed sensor is the combination of four materials of Cu, Au, rGO, and PEDOT:PSS to fabricate the bioelectronics sensor. The use of PEDOT:PSS more sensitized by rGO has improved the sensor performance in terms of limit detection and sensitivity and the incorporation of the metals Au and Cu has enhanced the electro-catalyst activity for glucose oxidation producing more electrons and providing faster transmission through the semiconductor (rGO/PEDOT:PSS). The sensor has been fabricated with a simple and cost effective (drop cast) method which is much simpler and cheaper than traditional electrochemical methods but with a competitive performance. A small amount (only 1 µL) of glucose solution is enough for the sensor to respond accurately.

## Results and discussion

### Sensor design and sensing mechanism

**Figure 1:**
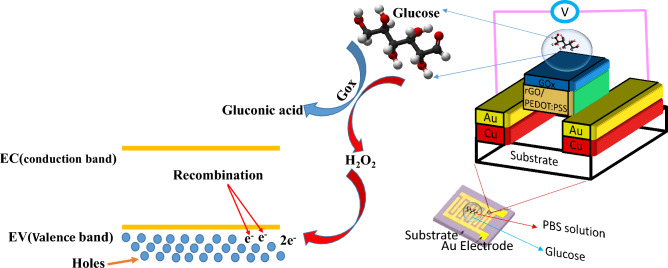
Schematic of the proposed bio-electronic biosensor based on rGO/PEDOT:PSS and its sensing mechanism.

We present and fabricate a bio-electronic biosensor which works based on electron transfer in semiconductors (p and n-type). The schematic of the proposed biosensor shown in Fig. 1 is a very high-potential alternative for other types of electrochemical methods commercial in the market. The proposed biosensor is a bio-electronic device that can be integrated into other biomedical devices. The electrical measurements do not need a potentiostat instrument but instead a resistive biasing circuit can perform the electrical measurement which has a much lower cost than the potentiostat device.

The sensing mechanism of the glucose sensor can be described as follow:

1 $${\text{D}} - {\text{Glucose}} + {\text{H}}_{{2}} {\text{O}} + {\text{O}}_{{2}} \to {\text{D}} - {\text{Gluconicacid}} + {\text{H}}_{{2}} {\text{O}}_{{2}}$$

2 $${\text{H}}_{{2}} {\text{O}}_{{2}} \to {\text{O}}_{{2}} + {\text{2H}}^{ + } + {\text{2e}}^{ - }$$

When the glucose oxidase reacts with the glucose molecules, as a result of this reaction, hydrogen peroxide is released. Under a bias voltage, hydrogen peroxide is separated into electrons^1,24^. In the following, the released electrons recombine with the majority carriers of holes. Increasing the concentration of glucose, more holes are recombined and finally, the conductivity of the sensor decreases. Figure 1 shows the sensing mechanism of the glucose bio-sensor based on Cu/Au/rGO/PEDOT: PSS.

There are two types of contributors to the fast response of the sensor: the chemistry and the electronic.

The materials used in the sensor provide high electrocatalytic activity for glucose oxidation. The metals used in this work, Au and Cu enhance the electro-catalyst activity for glucose oxidation producing more electrons in a shorter time. Also the PEDOT:PSS was combined with rGO and the concentration of rGO was optimized in sensor which led to a low response time. The rGO itself has high electrocatalytic activity for glucose oxidation^20,53^.

The contribution from electronics is based on higher mobility and lower resistance of rGO. We optimized the concentration of GOx as well which produced more electrons and these electrons could be transferred to the electrodes faster through graphene (due to its ballistic conduction).

### Structural and morphological properties of material

**Figure 2:**
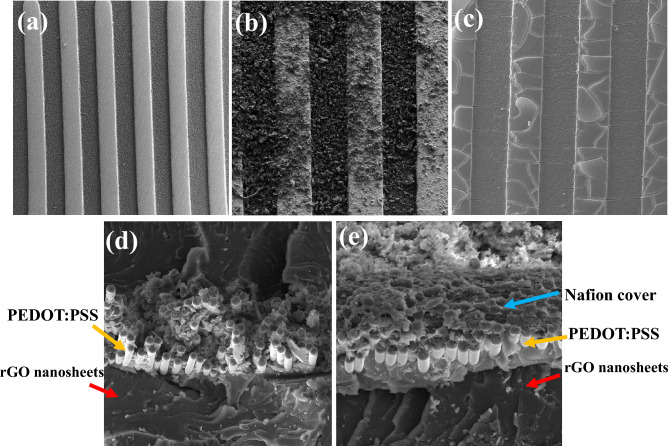
SEM image of (**a**) interdigital electrodes with 500 µm magnification. (**b**) graphene nanosheets on the gold electrodes. (**c**) PEDOT: PSS biosensor. (**d**) cross-sectional view of PEDOT: PSS/rGO layer (**e**) cross-sectional view of PEDOT: PSS/rGO/Gox/Nafion sensor.

The surface morphology of interdigitated electrodes and rGO/PEDOT: PSS layers were investigated by Scanning Electron Microscopy (SEM) model (TESCAN Mira3 device) and atomic force microscopy (AFM) model (Naio Afm) Device brand (NanoSurf). As you can see, the deposited materials on the IDE substrate are photographed step by step. As shown in Fig. 2a, at a magnification of 500 µm, the distance between the combs and the width of the combs are about 170 µm, respectively. Figure 2b shows the rGO layer deposited on IDE at a magnification of 200 µm. As it is represented the rGO material covered the electrodes. Figure 2c shows the SEM image of the biosensor based on PEDOT: PSS with a magnification of 200 μm. The PEDOT: PSS based sensor is fabricated on gold electrodes. The cross-sectional SEM image of rGO/PEDOT: PSS is represented in Fig. 2d. As it is shown PEDOT: PSS rods are placed on the rGO nanosheets. The cross-sectional view of the PEDOT: PSS/rGO/Gox/Nafion sensor is shown in Fig. 2e. It is obvious that Gox and nafion are immobilized on the surface of PEDOT: PSS rods and graphene nanosheets.

**Figure 3:**
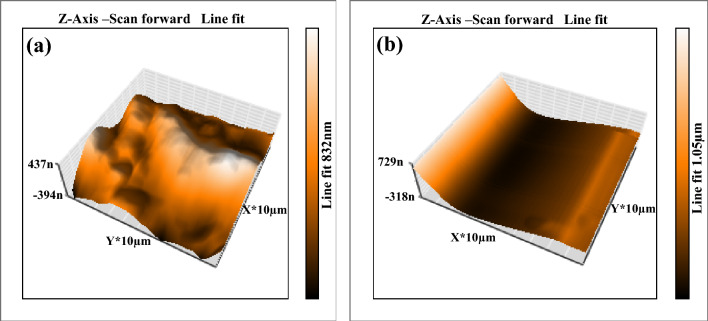
AFM image (**a**) before and (**b**) after enzyme immobilization.

Figure 3 shows the AFM image of the PEDOT: PSS/rGO layer before (Fig. 3a) and after (Fig. 3b) enzyme immobilization. The AFM images indicate changes in the morphology of the PEDOT: PSS/rGO before and after the Gox immobilization. The thicker layer shown after the immobilization is due to the adsorption of GOx on the surface of the PEDOT: PSS/rGO. Figure 3b presents more smooth edges of the PEDOT: PSS, indicating the successful immobilization of Gox.

### Electrical characteristics

To investigate the effect of rGO on the fabricated biosensor, we compared the electrical response of the PEDOT: PSS and the rGO/PEDOT: PSS as sensitive materials to glucose. The electrical characteristic of the fabricated bio-electronic glucose sensor was measured with a source management instrument model (Keithley 2450 Source Meter). A DC voltage is applied to the biosensor and the current is measured and recorded.

### PEDOT: PSS and rGO/PEDOT: PSS biosensor

**Figure 4:**
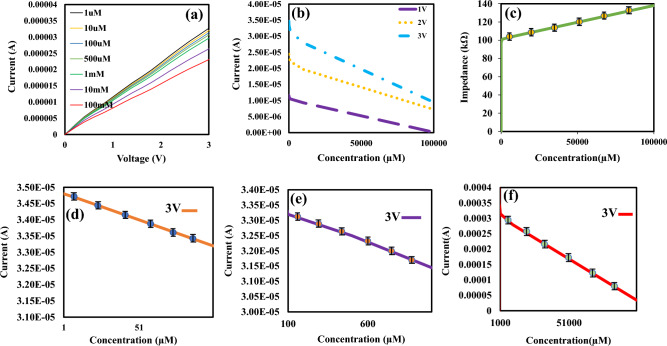
The electrical characteristic of the resistive bio-sensor based on PEDOT: PSS in the presence of glucose from 10 µM to 100 mM. (**a**) (I–V) curves for different glucose concentrations, (**b**) (I–n) curves for different applied voltages, (**c**) Impedance curve as a function of glucose concentrations (**d**) (I–n) curve for the applied voltage of 3 V for concentrations of 1 µM to 100 µM. (**e**) (I–n) curve for the applied voltage of 3 V for concentrations of 100 µM to 1 mM. (**f**) (I–n) curve for the applied voltage of 3 V for concentrations of 1 mM to 100 mM.

At first, we obtained the electrical responses with biosensors based on PEDOT: PSS. The voltage was swept from 0 to 3 V and the current was recorded when the fabricated biosensor was exposed to various glucose concentrations from 1 µM to 100 mM in PBS solution. The characteristic curves (I–V) is shown in Fig. 4. According to Fig. 4a, the current of the biosensor increases with the increment of the voltage for all glucose concentrations. The electrical characteristic (I–n) of the biosensor for various glucose concentrations (n) is plotted at voltages with values of 1, 2 and 3 V in Fig. 4b. From Fig. 4b, it can be seen that the sensor has the highest sensitivity at V = 3 V because slope of (I–n) curve is more than another voltage. In our sensor, the current of the biosensor increases with the increase of the voltage, which is almost a linear characteristic. Thus, the impedance of the biosensor is approximately constant, whose value is directly related to the concentration of glucose. Figure 4c show the impedance of biosensor toward to different glucose concentrations. According to Fig. 4c, the impedance of biosensor increases with the increase of the glucose. For more analysis, we divided the wide detection range of the sensor into three areas and examined the graphs of the glucometer construction at three different glucose levels. Concentration range: (1–100 μM) Fig. 4d, (100 μM to 1 mM) Fig. 4e and (1–100 mM) Fig. 4f. We observed a relatively linear response for all ranges.

**Figure 5:**
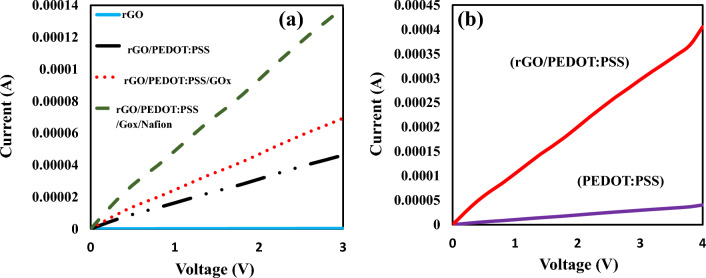
(**a**) (I–V) curves of each step of fabrication of rGO/PEDOT: PSS. (**b**) Biosensor response to 1 mM of glucose for PEDOT:PSS and rGO/PEDOT:PSS modes.

In this part the glucose sensitive material in the designed biosensor is rGO/PEDOT: PSS. In order to check electrical properties at each step of fabrication, the current–voltage (I–V) curve is taken from the biosensor output. According to Fig. 5a, it can be seen, after adding each material to rGO (PEDOT: PSS, Gox and Nafion), the electrical conductivity of the sensor is improved. PEDOT: PSS greatly increases the conductivity of the sensor due to its conductivity property. Next, by adding an enzyme of Gox, it reduces the conductivity due to creating a barrier in the path of the movement of electrons, indicating that the resistance of the sensor increases once the enzyme is introduced to the rGO/PEDOT:PSS surface. At the end of adding Nafion, it increases the conductivity of the sensor again.

**Figure 6:**
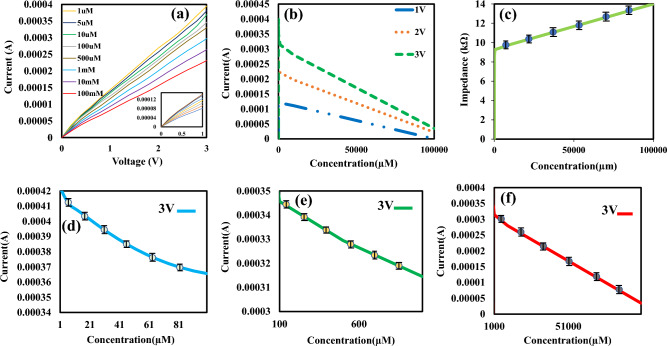
The electrical characteristic biosensor based on rGO/PEDOT: PSS in the presence of glucose from 1 µM to 100 mM. (**a**) (I–V) curve for various glucose concentrations, (**b**) current as a function of various glucose concentrations for different voltages, (**c**) Impedance curve as a function of glucose concentrations (**d**) (I–n) curve for the applied voltage of 3 V for the concentration of 1 µM to 100 µM. (**e**) (I–n) curve for the applied voltage of 3 V for the concentrations of 100 µM to 1 mM. (**f**) (I–n) curve for the applied voltage of 3 V for the concentrations of 1 mM to 100 mM.

The electrical characteristic of the proposed biosensor based on rGO/PEDOT: PSS/GOx/Nafion was investigated. To analyze the effect of the voltage on the overall performance of the biosensor based on rGO/PEDOT: PSS, the applied voltage was swept from 0 to 3 V and the current of the biosensor was measured within the presence of glucose for the concentrations of 1 µM to 100 mM in PBS solution as shown in Fig. 6a. Based on the results shown in Fig. 6a, the whole current will decrease by increasing the concentration of glucose. From Fig. (6b), it can be seen that the sensor is the most sensitive to V = 3 V (slope of (I–n) curve is more than another voltages). We measured and plotted the impedance curve as a function of glucose concentration in Fig. 6c. According to Fig. 6c, the impedance of biosensor increases with the increase of the glucose. We divided the widespread sensor detection range into three regions for a more accurate analysis, and the diagrams for the glucometer structure in three different glucose concentrations. We did: (1–100 μM) Fig. 6d, (100 μM to 1 mM) Fig. 6e and (1–100 mM) Fig. 6f. It is presented that rGO/PEDOT: PSS sensor is more sensitive than PEDOT: PSS which is discussed in detail in the sensitivity section.

For each fabricated sensor device, the following parameters are investigated to evaluate the property of the sensor: Sensitivity, Selectivity, Stability, Repeatability, and Reproducibility.

**Figure 7:**
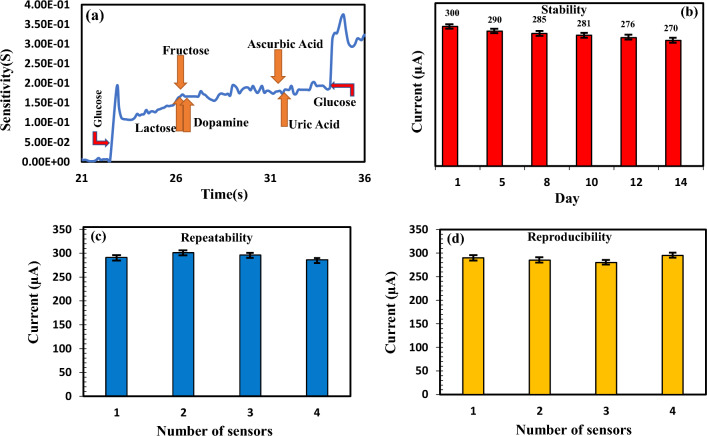
The plots of (**a**) selectivity, (**b**) stability, (**c**) repeatability, and (**d**) Reproducibility of biosensor based on rGO/PEDOT: PSS.

In order to investigate the selectivity of the proposed biosensor, its responses have been examined within the presence of glucose and interfering species such as ascorbic acid (65 µM), uric acid (0.34 mM), lactose (1.34 mM), fructose (4.4 mM), and dopamine (100 pM). The selected concentrations of interfering species are in the range of their concentrations in human blood. Figure 7a shows the selectivity of the proposed biosensor. As shown in Fig. 7a, first we poured the glucose solution which caused the sensor output to increase. Then we poured the interfering species and we observed no change in the output current. Finally, we poured the glucose solution again, which lead to a rise in the sensor response output. This indicates that the designed biosensor responds to glucose very sensitively but does not respond to interfering species.

The stability of the fabricated biosensor has been performed through one biosensor for two weeks. The biosensor confirmed moderate stability for cycles of measurements and retained 90% of the preliminary response value after two weeks as shown in Fig. 7b.

The repeatability was tested by four instances of measurements through the same sensor for 5 mM of glucose concentration as proven in Fig. 7c. RSD of the repeatability has been obtained about 0.8%, showing good repeatability of the biosensor.

The reproducibility of the proposed biosensor was examined for four sensors with the same preparation procedure and calculated in response to 5 mM of glucose concentration. The currents of biosensors have been calculated in the presence of glucose concentration and have been compared. The currents are shown in Fig. 7d. The RSD of the reproducibility was calculated about 0.91%.

### Evaluation parameters

A sensor’s sensitivity indicates how much its output changes when the input quantity changes. Sensitivity is measured as follows:

3 $$S = \frac{{\Delta {\text{I}}}}{{\Delta {\text{n}}}} = \left| {\frac{{{\text{In }} - {\text{I}}_{0} }}{{\Delta {\text{n}}}}} \right|$$

where I_n_ and I_0_ are the currents in the presence and the absence of glucose solution, respectively which are extracted from (I–n) curves. $\Delta$n is the difference in glucose concentration. Based on the results shown in Figs. 4b and 6b, the difference in currents for successive glucose concentrations increases by increasing the voltage, it can be concluded that the sensitivity of the biosensor improves by applying higher voltage. The sensitivities for rGO/PEDOT: PSS and PEDOT: PSS biosensors have been calculated and compared. The sensitivity of the biosensor based on PEDOT: PSS (S_1_) towards glucose has been calculated 8.54 × 10^–2^ μA μM^−1^. The sensitivity of the rGO/PEDOT: PSS (S_2_) has been calculated 5.65 μA μM^−1^ from 0 to 100 µM of glucose with a detection limit of 1 µM the basis of the following Eq. (3). Biosensor response for 1 mM of glucose concentration for PEDOT:PSS and rGO/PEDOT:PSS modes are plotted in Fig. 5b. According to Fig. 5b, the current will increase by the adding rGO to PEDPT: PSS. So, it is shown that rGO improves the sensitivity of the sensor. Based on results, the use of biosensor based on rGO/PEDOT: PSS in the proposed electronic biosensor has advantages such as higher sensitivity and performance compared to biosensors based on PEDOT: PSS.

The sensitivity of the biosensors for different detection ranges at 3 V, which is the optimal voltage was calculated and listed in Table 1, which according to the calculated numbers shows us that the sensitivity of rGO/PEDOT:PSS is more and better than PEDOT:PSS.

**Table 1:** Calculated sensitivity of biosensors based on PEDOT: PSS and rGO/PEDOT: PSS at 3 V.

Type of sensitivity	Detection linear range	Sensitivity (μA μM^−1^)
S_1_	1 to 100 µM	8.54 × 10^–2^
	100 µM to 1 mM	1.94 × 10^–3^
	1 mM to 100 mM	3.88 × 10^–5^
S_2_	1 to 100 µM	5.65
	100 µM to 1 mM	3.45 × 10^–2^
	1 mM to 100 mM	3.88 × 10^–4^

### Blood serum sample tests

Five blood serum samples were taken and measured by the commercial device in the pathology laboratory from Shahid Beheshti Hospital. The five serum samples have been used with different glucose values such as 4.29, 5.29, 6.02, 7.80, and 12.65 mM. The values obtained with the commercial device and values obtained with our glucometer were compared and further analyzed as listed in Table2. Based on the result in Table 2, the fabricated biosensor can be used as a great method for glucose detection in blood real serum samples and the values are very agreement with the commercial device.

**Table 2:** Comparison of glucose values obtained by the commercial device in a laboratory with the values measured by the rGO/PEDOT: PSS -based biosensor.

Values measured by commercial device	Values measured by our biosensor	RSD (%)	Recovery (%)
4.29 mM	4.31 mM	0.46	99.99
5.29 mM	5.26 mM	0.56	100.005
6.02 mM	6.04 mM	0.33	99.99
7.80 mM	7.74 mM	0.77	100.007
12.65 mM	12.55 mM	0.79	100.007

### Real human blood tests

Tests of six blood samples were taken and measured by a commercial device in the pathology laboratory of Shahid Beheshti Hospital. Six blood samples with different amounts of glucose such as 78, 81, 80, 84, 315, 380 mg/dl have been used. The values obtained with the commercial device and the values obtained with our glucometer were compared and further analyzed as listed in Table 3. The results showed that our glucometer has high clinical accuracy as shown in Table 3. In order to check the reproducibility of the other test strips, we immediately performed additional tests to confirm that changes in blood glucose did not alter the false test criteria. Also, to check the repeatability of the tests, each test was performed three times to ensure the accuracy of the results. During the experiments, blood glucose test results were recorded. The obtained data showed that our test strips have excellent performance with high clinical accuracy.

**Table 3:** Comparison of blood glucose values of samples obtained by a commercial device and our glucometer.

Values measured by commercial device	Values measured by our glucometer	RSD (%)	Recovery (%)
81 mg/dL	83 mg/dL	0.024	99.97
80 mg/dL	79 mg/dL	0.012	100.01
78 mg/dL	76 mg/dL	0.025	100.02
84 mg/dL	86 mg/dL	0.023	99.97
315 mg/dL	312 mg/dL	0.0956	100.009
380 mg/dL	383 mg/dL	0.0786	99.99

Some recently reported glucose sensors have been compared with the responses obtained from our Cu/Au/rGO/PEDOT: PSS sensor in Table 4. Based on Table 2, the fabricated glucose biosensor indicated a high sensitivity (5.65 μA μM^−1^), a low detection limit of 1 µM and, a fast response which shows its impressive potential in the detection of wide glucose concentrations (0–100 mM). The results obtained in this work showed that the fabricated biosensor can be used as an alternative to the electrochemical sensors. Its stabilized fabrication process makes it an excellent selection for lab on chip application. It can also be used as portable and wearable blood glucose sensor.

**Table 4:** Comparing several similar works in different years with this work.

Sensing material	Sensitivity	Detection time	LDR	DL	Year	References
CuO/graphene	37.63 µA mM^−1^ cm ^−2^	5 s	5–14 mM	5 µM	2018	^50^
GCE/ZnO.NDCS/GOx	231.7 µA mM^−1^ cm^−2^	5 s	0.2–12 mM	6.3 µM	2018	^51^
Pd/Graphene	3%	5 s	1–10 µM	1 nM	2018	^52^
Graphene/Ag	9.9596 μA/μM	8 s	0.1–0.35 μM	0.0262 μM	2020	^54^
Graphene	nr	5 s	0.05–100 mM	0.15 μM	2020	^55^
CuO/ZnO	6.643 mV/mM	6 s	1–8 mM	1 mM	2021	^56^
PEDOT:PSS	43.52 μA mM^−1^ cm^−2^	4 s	0.05–0.5 mM	0.05 mM	2022	^57^
Ni(OH)2/Fe2O3	24.59 μW cm^–2^ mM^–1^	5 s	0.05–0.25 mM	0.05 mM	2022	^58^
rGO/PEDOT:PSS	5.65 mA mM^−1^	2 s	0–100 mM	1 µM	2023	This work

**Figure 8:**
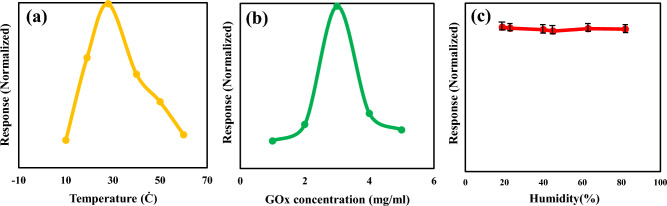
Response of biosensor with respect to (**a**) temperature (**b**) Gox concentration and (**c**) humidity for 5 mM glucose.

To fabricate a high-performance biosensor for glucose detection, the effect of humidity, temperature and enzyme concentration on the response of rGO/PEDOT: PSS based biosensor was investigated. The effect of temperature on the performance of biosensor was evaluated by varying the temperature from 10 to 61 °C in Fig. 8a. The vertical axis is defined as response (R):

4 $$R = \left| {\frac{{{\text{In}} - {\text{Io}}}}{{\text{Io }}}} \right|$$

where I_o_ and I_n_ are the current of the biosensor in the presence and the absence of glucose, respectively. Since, the enzymes are sensitive to temperature, the equation curve of enzyme as a function of temperature is exponential. As shown in Fig. 8a, the response of our sensor gradually enhances with increasing temperature up to ~ 27 °C. The incensement of current response with increased temperature is due to the improved enzymatic performance and a decrease in dissolved O_2_ value. With the increment of temperature, the enzyme’s chemical reaction and its kinetic energy also increase to oxidation of glucose. Finally, leads to the increased amount of glucose concentration to oxidation and increment of electrons. Therefore, the sensor response is improved. A drastic drop in the current response is observed from 27 to 40 °C. Beyond 27 °C, the current response decreases because of the heating effect of the immobilized enzyme that decreases the enzyme’s rate of reaction due to the GOx molecule that denatures. So, a constant temperature of 27 °C has been selected for all measurements. To investigate the effect of the GOx molecules for immobilization, its concentration was changed from 1 to 5 mg/mL in response to 5 mM glucose. As shown in Fig. 8b, the increment of the enzyme leads to an improvement of the chemical reaction rate. However, this glucose concentration had an effect only up to a certain concentration depending on the glucose molecule concentration. As shown in Fig. 8b, the current response enhances with increasing the amount of enzyme and peaked at 3 mg/mL. According to above discussed results the kinetically limited reaction of GOx, wherein oxygen and glucose consumption was directly proportional to GOx value. With the increased amount of enzyme, an increased number of free glucose molecules to oxidation, results in an increased current response. Further increases in GOx value do not change the response as the oxidation is limited by diffusion because not all enzymes attend in the reaction. The same result as that of Ang et al. was obtained. The influence of relative humidity on the behavior of GOx immobilized onto rGO/PEDOT: PSS was carried out by current response with different relative humidity values as shown in Fig. 8c. The response of the sensor was measured at a humidity of approximately 18% to 84% during one day with one device. Figure 8c indicates that the biosensor can maintain its sensing performance in humidity conditions.

### Portable smart glucometer design

**Figure 9:**
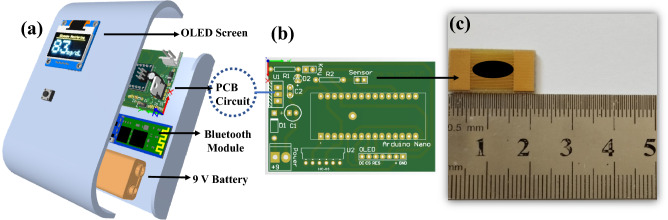
(**a**) The schematic of the proposed glucometer, (**b**) The schematic of the electronic board designed in the Altium Designer program, (**c**) the real image of fabricated glucose sensor.

A portable smart glucometer for practical blood glucose monitoring is designed. The glucometer integrates an OLED screen for glucose value display in the unit of mg/dl, and a microcontroller for signal processing. A 9 V battery as a power supply, and a glucose sensor connect to the glucometer to detect glucose levels. A mobile application and website were developed to receive blood glucose values and display them on a smartphone. Using this technology, we believe our glucometer could provide tranquility in daily life for diabetic patients and help with health care. Figure 9a shows the fabricated glucometer device. The schematic of the circuit designed in the Altium Designer program is presented in Fig. 9b and a real photo of glucose sensor is shown in Fig. 9c.

### Website and mobile application design

**Figure 10:**
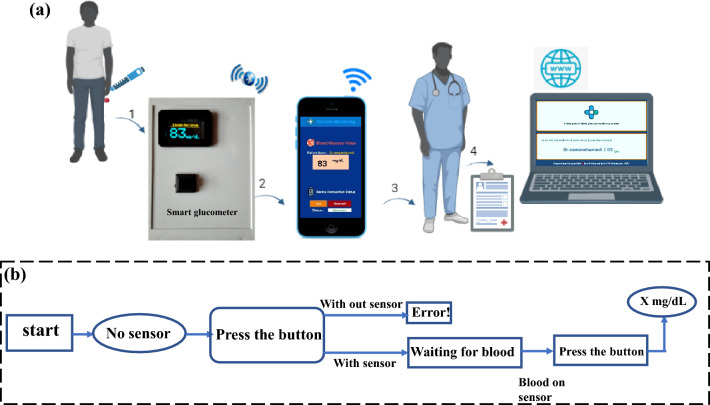
(**a**) The schematic of developed portable smart glucometer. (**b**) The designed glucometer working algorithm.

The application of mobile provides users to read and check the monitored blood glucose value. Also, the application enables storing historic values and plotting the glucose level. The statistics are sent to android phone in a serial shape. The benefit of using the Bluetooth module is that the specialist can watch the patient's blood glucose value from a separate part. To improve this project into greater commonsense, for displaying the statistics on a smartphone, the facts are despatched to an online website with HTML, CSS, and PHP programming dialects. The method of sending facts is using the HTTP conference and harbor 80. In this way, the specialist doctor can observe, manipulate and deal with the patient's blood sugar from anywhere inside the global who has got to the Web. Also, at each second the blood sugar value can be transferred to the website online. Figure 10a indicates the schematic of the developed portable and smart glucometer, the blood sugar value is visible on the OLED display, through the android application on the smartphone, and additionally through the website anywhere in the world. The working algorithm of the designed glucometer is shown in Fig. 10b. The name of the Android application (Glucose_Monitoring) which you can visit the site (monfared-lab.ir) to download.

To compare the fabricated glucometer with other available electrochemical glucometers, the parameters like the range of detection, response time and type of enzyme are listed in Table 5. Table 5 indicates our glucometer has a wider detection range, faster response time, and an acceptable cost for per sensor than other commercial glucometers.

**Table 5:** Comparison of manufactured glucometer and other commercial electrochemical glucometers.

Glucometer brand	Range (mg/dl)	Response time	Type of enzyme	Cost of per sensor
FreeStyle Freedom Lite	20–500	5 s	GDH-FAD	$0.26
AgaMatrix JAZZ	20–600	5 s	GOx	$0.33
Accu-Chek Aviva Plus	20–600	5 s	GDH-PQQ	$0.69
OneTouch Ultra2	20–600	5 s	GOx	$0.76
Accu-Chek Nano	20–600	4 s	Mut Q-GDH	$0.99
SideKick	20–600	8 s	GOx	$0.35
Electrode based rGO/PEDOT:PSS	0.178–1780	2 s	GOx	$0.2

## Conclusion

In summary, we fabricated a portable smart glucometer with high accuracy. A sensor patch allows glucose detection, ensures comfortable and facilities glucose monitoring. The Cu/Au/rGO/PEDOT: PSS hybrid structure was used as the bioelectronic glucose sensor to improve the response.

When the glucose oxidase reacts with the glucose molecules, hydrogen peroxide is released. Under a bias voltage, hydrogen peroxide is separated into electrons. So, the released electrons recombine with the majority carriers of holes. Increasing the concentration of glucose, more holes are recombined and finally, the conductivity of the sensor decreases. The concentration of glucose was detected in a range from 0 to 100 mM that covered glucose values in diabetic patients and healthy people with a very low detection limit of 1 µM. The fabricated Cu/Au/rGO/PEDOT: PSS showed excellent sensing performance such as high selectivity, high sensitivity and good stability. The sensitivity of the biosensor towards glucose was calculated at about 5.65 µA mM^−1^. The glucometer has been tested on 11 samples revealed high clinical accuracy of glucose measurements in human blood, indicating worth further improvement for PoC (point of care) applications. The PCB could be miniaturized and integrated into existing glucometer models to fabricate a true glucose monitoring glucometer.

## Methods

### Human Blood and serum samples

Blood and blood serum samples were purchased from Shahid Beheshti Hospital. All experiments and methods were performed in accordance with relevant guidelines and regulations. The experimental protocols were approved by the pathology department of Shahid Beheshti Hospital,Shiraz, Iran.

All procedures performed in this study were in accordance with the ethical standards of the laboratory of Shahid Beheshti Hospital. Informed consent was obtained from all subjects.

### Material and apparatus

All chemicals were of analytical reagent grade. D (+) glucose (97%), glucose oxides (GOx, EC 1.1.3.4, type VII from Aspergillus Niger, 221 U mg^−1^), nafion (5% solution), buffer solution phosphate (PBS), reduced graphene oxide (rGO), ethanol (96%), were purchased from Sigma Aldrich. Poly (3,4- ethylenedioxythiophene): poly (4-styrenesulfonate) (PEDOT: PSS) ink in water solvent with 1.3–1.7 (wt %) solid content was purchased from Ossila. To prepare the sensitive material of the biosensor, 100 mg of rGO was dissolved in 10 mL of water and sonicated for 20 min. The concentration of PEDOT:PSS was 1 mM. Nafion was mixed with ethanol (5% solution in 96% ethanol) with 1:1 (wt %) and stirred for one minute. The concentration of Gox was 3 mg/mL. 3 mg of GOx was dissolved in 1 mL of PBS (0.01 M, pH 7.4)). The used PBS solution has the properties of 0.01 M and, pH 7.4.

### Fabrication of sensor

**Figure 11:**
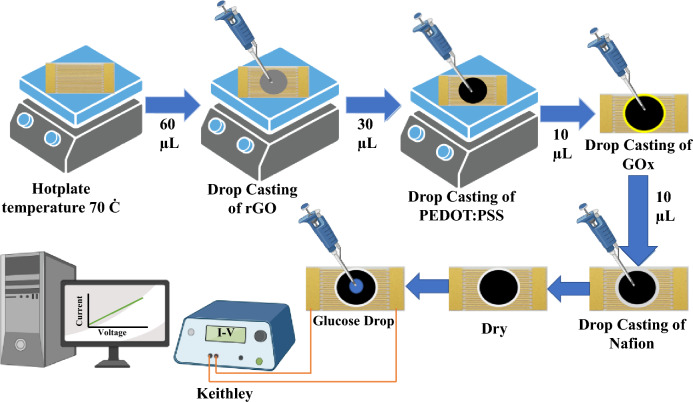
Schematic of the fabrication process of the proposed bio-sensor.

We have fabricated two types of sensors based on PEDOT: PSS and rGO/PEDOT: PSS materials that are deposited on IDE substrate for blood glucose sensing. We have measured and compared their electrical responses. The fabrication process of the biosensor is schematically shown in Fig. 11. The interdigitated electrodes were patterned by lithography on a PCB with a 1 × 2 cm^2^ area containing 30 fingers, 170 µm gap spacing, and 170 µm finger widths. To make the microelectrodes, 100 nm Cu and Au was deposited on the PCB substrate using sputtering. In order to fabricate the biosensor, a three-step process is used for cleaning interdigitated electrodes. The electrodes are first brush scrubbed in an aqueous solution. In the second step, they are ultrasonicated in an aqueous bath. In the final step to enhance the cleaning effect of electrodes, the electrodes are cleaned with ethanol and acetone in an aqueous bath. To prepare the biosensor based on rGO/PEDOT: PSS, the substrate has been placed on the hotplate at 70 °C and then 60 µL of the rGO solution was drop cast on the IDE substrate. In the following, 30 µL of PEDOT: PSS solution (1 mM) was sonicated for 5 min and dropped on rGO layer. For the immobilization of GOx, 3 mg of GOx was dissolved in 1 mL of PBS (0.01 M, pH 7.4) and 30 μL of the prepared GOx solution was dropped on the surface of rGO/PEDOT: PSS thin film and allowed to dry at room temperature for about 30 min. After that the device was kept at 4 ºC for 12 h and then 10 μL Nafion was dropped on the surface of deposited materials. Finally, a biosensor based on rGO/PEDOT: PSS/Gox /Nafion was fabricated, successfully. After each step of fabrication, the current–voltage (I–V) curve is obtained from the biosensor output. The above fabrication process has been repeated for biosensors based on PEDOT: PSS. Figure 11 shows the schematic of the proposed biosensor manufacturing process.

## Data Availability

The datasets generated and analyzed during the current study are available from the corresponding author on reasonable request.
